# Tuning the Thermoresponsive Behavior of Surface-Attached PNIPAM Networks: Varying the Crosslinker Content in SI-ATRP

**DOI:** 10.1021/acs.langmuir.0c03545

**Published:** 2021-03-15

**Authors:** Sophia Thiele, John Andersson, Andreas Dahlin, Rebekah L. N. Hailes

**Affiliations:** Department of Chemistry and Chemical Engineering, Chalmers University of Technology, 41296 Gothenburg, Sweden

## Abstract

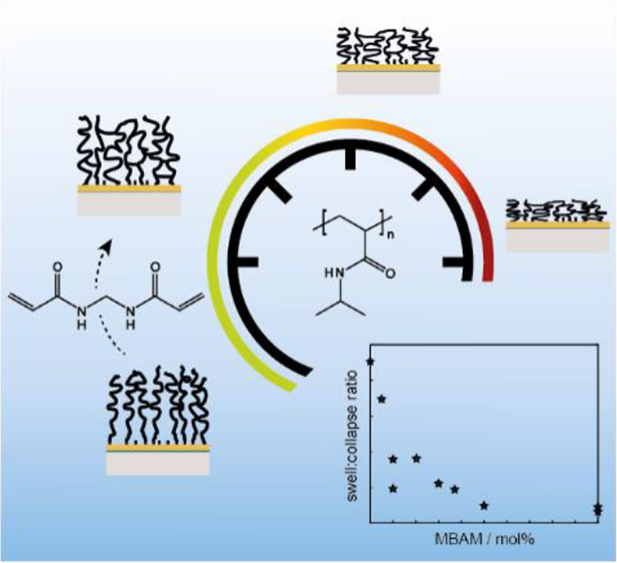

The synthesis and thermoresponsive properties of surface-attached poly(*N*-isopropylacrylamide)-*co*-*N*,*N*′-methylene bisacrylamide (PNIPAM-*co*-MBAM) networks are investigated. The networks are formed *via* SI-ARGET-ATRP (“grafting-from”) on thiol-based initiator-functionalized gold films. This method is reliable, well controlled, fast, and applicable to patterned surfaces (*e.g.*, nanopores) for networks with dry thicknesses >20 nm. Surface-attached PNIPAM-*co*-MBAM gels are swollen below their volume phase transition temperature but above collapse without complete expulsion of water (retain ∼50 vol %). The swelling/collapse transition is studied using complementary SPR and QCMD techniques. The ratio between swollen and collapsed heights characterizes the thermoresponsive behavior and is shown to not depend on network height but to vary with MBAM content. The higher the proportion of the crosslinker, the lower the magnitude of the phase transition, until all responsiveness is lost at 5 mol % MBAM. The temperature range of the transition is broadened for more crosslinked PNIPAM-*co*-MBAM gels but remains centered around 32 °C. Upon reswelling, less crosslinked networks display sharp transitions, while for those containing ≥3 mol % MBAM, transitions remain broad. This tunable behavior persists for gels on nanostructured gold surfaces. Investigating PNIPAM-*co*-MBAM networks on gold plasmonic nanowell arrays is a starting point for expanding their scope as thermo-controlled nanoactuators.

## Introduction

Poly(*N*-isopropyl acrylamide) (PNIPAM) is a particularly well-studied thermoresponsive polymer due to its biocompatibility and biologically relevant lower critical solution temperature (LCST) of ∼32 °C in aqueous media (*i.e.*, close to physiological temperature).^1,2^ Above this critical solution temperature, the unfavorable entropic contribution from the hydrophobic effect to the free energy of mixing dominates over exothermic hydrogen bonding, decreasing the solvent quality of water to the point where the polymer undergoes a sharp transition from a hydrated, extended coil conformation to a hydrophobic, collapsed structure (which maximizes intra- and interchain interactions). By exploiting these responsive properties, PNIPAM hydrogels have been used in drug delivery systems,^3^ cell culturing,^4^ and artificial muscles.^5^ PNIPAM brushes, formed by end-tethered chains on a variety of surfaces, have served as thermo-controlled nanoactuators (such as valves,^6^ pumps,^7^ and filters^8^) in microfluidic devices.^9,10^ The extent of the thermally induced collapse of the polymer brushes depends on grafting density and molecular weight,^11,12^ both of which can be controlled by surface-initiated (also termed “grafting-from”) atom transfer radical polymerization (SI-ATRP).^13−22^ The LCST is known to remain essentially the same as for coils in solution (±1 °C) regardless of the grafting strategy.

The magnitude of the thermoresponsive transition can be controlled by introducing crosslinks between polymer chains. For nonresponsive gels on substrates, crosslinkers significantly diminish the extent to which networks can swell.^23−25^ This has been exploited to vary the mechanical and swelling properties of poly(acrylamide) (PAAM)^24^ and furthered to include pH-responsive polyelectrolyte brushes.^26^ Investigations into crosslinking PNIPAM brushes grafted from nanoparticles noted the change in volume phase transition temperature (VPTT), network mesh size, and permeability.^27^ To more comprehensively study the effect of crosslinker content on swelling behavior, Harmon *et al.* prepared PNIPAM gels *via* common free radical polymerizations prior to spin-coating on silica substrates.^28^ However, swelling factors varied even within samples containing the same proportion of crosslinker. This was thought to occur due to stress-induced orientation introduced to the PNIPAM networks in the spin-coating process. Additionally, surface-attached networks prepared *via* free radical polymerizations have been shown to have an inhomogeneous crosslinking density, especially compared to gels synthesized *via* a controlled/living radical polymerization techniques, for example, ATRP.^29^ Besides homogeneous crosslinking, ATRP provides further advantages in surface functionalization: the transfer agents used are commercially available and show a high functional group tolerance, and the reaction is applicable to various surfaces (patterned or smooth) by choosing appropriate initiators.^20^ To circumvent limits imposed by the oxygen-sensitive nature of ATRP, a more tolerant option dubbed “activators regenerated *via* electron transfer” ATRP (ARGET-ATRP) is often used.^30,31^ To the best of our knowledge, there are no studies on the influence of crosslinking on the swelling/collapsing behavior of PNIPAM brushes, partly as determining the accurate brush heights in the solution is challenging.^32,33^ It should be noted that the degree of swelling is expected to depend on the geometry and orientation of the chains. For instance, in a brush on a planar surface (compared to a gel in 3D), there is only one spatial dimension available for expansion.

In this work, we varied the amount of a covalent crosslinker, *N*,*N*′-methylene bisacrylamide (MBAM), and reported its influence on the thermoresponsive behavior of PNIPAM brushes prepared *via* surface-initiated ARGET-ATRP (SI-ARGET-ATRP) on planar and patterned gold surfaces. Taking advantage of the metallic support, heights in the solution above and below the characteristic VPTT were obtained from SPR measurements using our previously reported non-interacting probe method.^34^ Quartz crystal microbalance with dissipation monitoring (QCMD) experiments support these results and give further insights into the phase transition temperature range. We discuss our results in the context of physisorbed and spin-coated PNIPAM-*co*-MBAM gels of similar thicknesses,^28,35,36^ PNIPAM networks on nanoparticles,^27^ and other types of crosslinked polymer brushes grafted from planar substrates *via* ATRP.^24,25^

## Experimental Section

### Chemicals

All chemicals were purchased from Sigma-Aldrich and used as received unless stated otherwise. H_2_O_2_ (30%) was from ACROS, *N*-isopropyl acrylamide (NIPAM) and NH_4_OH (28–30%) from Fischer, H_2_SO_4_ (98%) and EtOH (99.5%) from SOLVECO, and ω-mercaptoundecyl bromoisobutyrate from ProChimia. Water was of ASTM research grade type 1 ultrafiltered water (Milli-Q water). Buffers were based on phosphate-buffered saline (PBS) tablets (0.01 M phosphate, 0.13 M NaCl, pH 7.4).

Monomer NIPAM (99%) was recrystallized from hexane at 85 °C and stored under N_2_. The polymerization solvent MeOH was dried over 3 Å molecular sieves (Merck) and then stored under N_2_.

### Surface Cleaning

Prior to surface functionalization, SPR and QCMD sensors were cleaned in piranha wash (H_2_SO_4_/H_2_O_2_, 3:1 v/v) for 30 min and then rinsed in Milli-Q. The sensors and nanowell-patterned surfaces were then cleaned in RCA1 wash (H_2_O/H_2_O_2_/NH_4_OH 5:1:1 v/v) at 80 °C for 30 min, rinsed in Milli-Q and EtOH, and then dried with N_2_.

### SAM Formation

Clean gold surfaces were immersed in a 3 mL EtOH solution containing the ATRP initiator ω-mercaptoundecyl bromoisobutyrate (3 μL, 2 mM) and shaken (50 rpm) for 18 h. After incubation, the substrates were rinsed in EtOH and dried under N_2_.

### SI-ARGET-ATRP

PNIPAM brushes and PNIPAM-*co*-MBAM gels were prepared under the same reaction conditions. Reactions were carried out using standard Schlenk line techniques under an inert atmosphere of N_2_. The amount of MBAM supplied in the monomer feed varied between 0 mol % (for brushes) up to 10 mol %. Depending on the desired crosslinker content, varying amounts of both monomers were used so that the total monomer concentration was always 0.96 M. An example synthesis of PNIPAM-*co*-MBAM_1%_ is described below.

In one flask, monomer NIPAM (1.076 g, 9.5 mmol, 792 equiv), crosslinker MBAM (14.8 mg, 0.096 mmol, 8 equiv), and MeOH (8 mL) were added to the inhibitor remover. The solution was degassed with N_2_ for 5 min. In a second flask, CuBr_2_ (2.7 mg, 0.012 mmol, 1 equiv) was added to PMDETA (26.7 μL, 0.128 mmol, 10.7 equiv). The monomer solution was filtered (0.2 μm PTFE syringe filter) into the second flask, and the light blue solution obtained was degassed for a further 20 min. Separately, ascorbic acid (8.5 mg, 0.048 mmol, 4 equiv) was added to MeOH (10 mL) and degassed for 20 min. Gold sensors with SAM were removed from the initiator solution, washed with EtOH, dried, and placed in a Schlenk flask. The light blue reaction solution was transferred to this flask *via* cannula. To initiate the polymerization, 2 mL of ascorbic acid solution was added. The reaction was then shaken (50 rpm), and an additional 1 μL min^–1^ ascorbic acid solution was continuously supplied *via* a syringe pump and a PTFE tube until the reaction was quenched by exposure to air. Finally, the sensors were rinsed with Milli-Q water and EtOH and dried.

### Measurements

Details of IR-RAS, QCMD, SPR, and nanoplasmonic measurements can be found in the Supporting Information.

## Results and Discussion

**Scheme 1:**
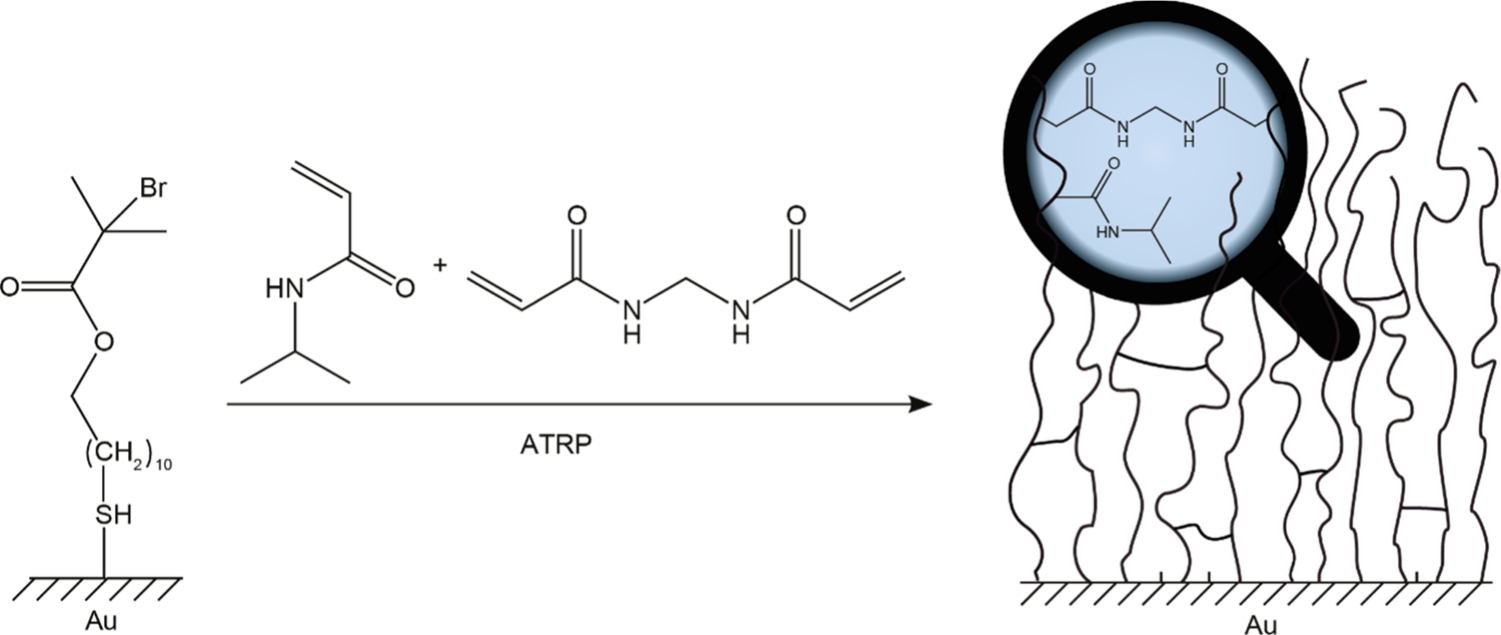
Chemical Structure of the ATRP Initiator on Gold, Reacting with PNIPAM and the Crosslinker,*N*,*N*′-Methylenebisacrylamide, to Give Brush Networks

PNIPAM-*co*-MBAM networks were prepared on gold surfaces initiated with an ω-mercaptoundecyl bromoisobutyrate self-assembled monolayer (SAM) *via* SI-ARGET-ATRP (Scheme 1), taking inspiration from previously described procedures.^27,30,37−41^ The chemical composition of networks with varying amounts of crosslinker were confirmed by infrared spectroscopy (Figure S1), which proved in line with the reported spectra.^42^ The appearance and growth of a band at 1725 cm^–1^ with increasing crosslinker content was attributed to a C=O stretch from MBAM and confirmed the incorporation of the crosslinker.

**Figure 1:**
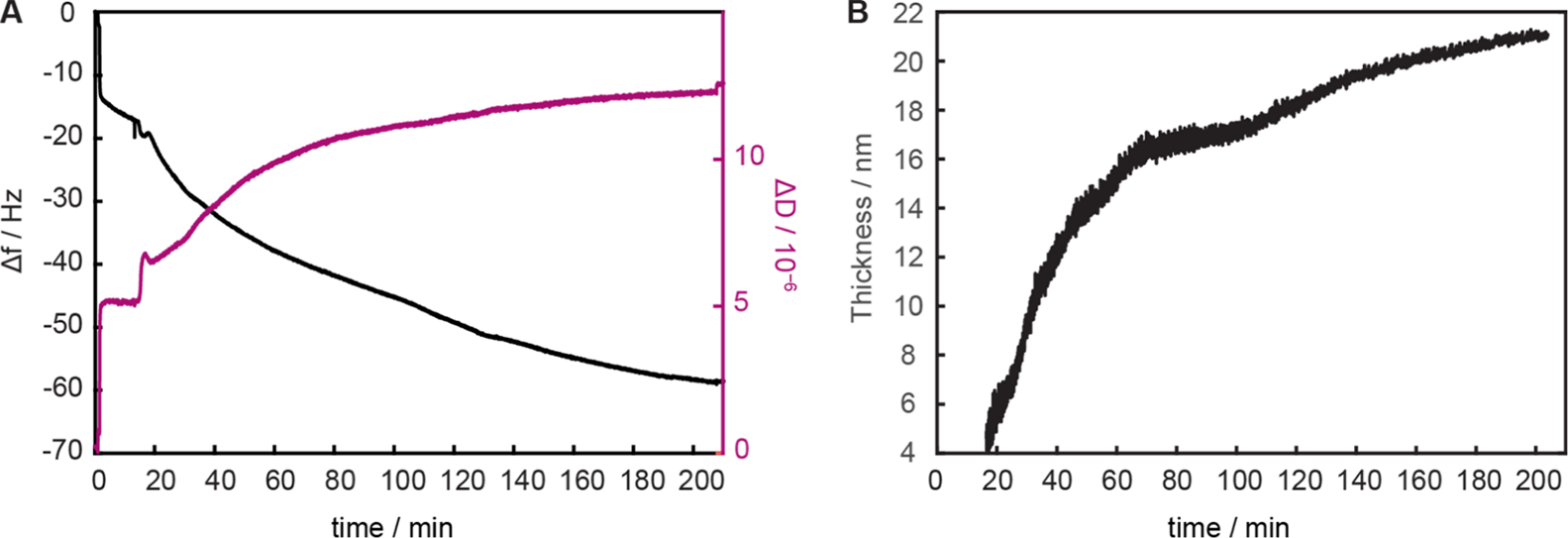
*In situ* ATRP (MeOH, 0.48 M) monitored in QCMD yields PNIPAM-*co*-MBAM_1%_. (A) Change in frequency and dissipation over time. (B) Layer thickness over time calculated by Voigt modeling and curve fitting of multiple frequency and dissipation overtones.

To study the kinetics of polymerization, reactions with an NIPAM/MBAM molar ratio of 99:1 (0.96 M) were carried out in methanol for up to 30 h at ambient temperature. The resulting polymer network heights were measured in air using surface plasmon resonance (SPR). The polymerization proceeded quickly initially: after 1 h, 28 nm-thick networks were observed. However, the gel thickness remained constant after this (up to 30 h, Figure S2), presumably due to the significant termination reactions caused by low local monomer concentration, radical combination, or catalyst loss, in line with what has been previously observed.^24,34^ For all reaction times, the SPR angle (θ_SPR_) and thus the dry height of any particular surface remained constant (Δdry_average_ = 1.0 nm) across two different positions (Table S2), indicating a homogeneous gel thickness. For better control over thin networks, the ATRP kinetics can be slowed by lowering the monomer concentration.^43^ Thus, an *in situ* polymerization of PNIPAM-*co*-MBAM_1%_ was monitored at a lower concentration (0.48 M) using QCMD (Figure 1A). The initial decrease in frequency (increase in coupled mass) and simultaneous increase in dissipation occur due to the formation of a viscoelastic layer, that is, polymerization initiation. This was followed by linear growth until ∼2 h. The dissipation starts to flatten after ∼1 h, indicating the formation of a more rigid layer compared to initially, potentially due to enhanced crosslinking. In general, given the relatively low-frequency signals, the flattening curves again point toward termination reactions, similar to more concentrated polymerizations as discussed above. While modeling viscoelastic layers is often challenging,^44,45^ we were able to apply Voigt-based models to quantitatively determine the layer thickness evolution by fitting changes in frequency and dissipation at multiple harmonics (Figure S3).^46−49^ A frequency-independent PNIPAM-*co*-MBAM layer density of 860 kg m^–3^ (20% polymer brush and 80% methanol) and standard methanol density and viscosity^50^ were assumed. The maximum gel thickness measured was 21 nm at 3.5 h (Figure 1B). This is lower than heights measured by SPR but is not unrealistic as despite measures to maintain an inert atmosphere, some inhibition due to oxygen is expected compared to *ex situ* polymerizations (*e.g.*, as PTFE tubing is permeable).

**Figure 2:**
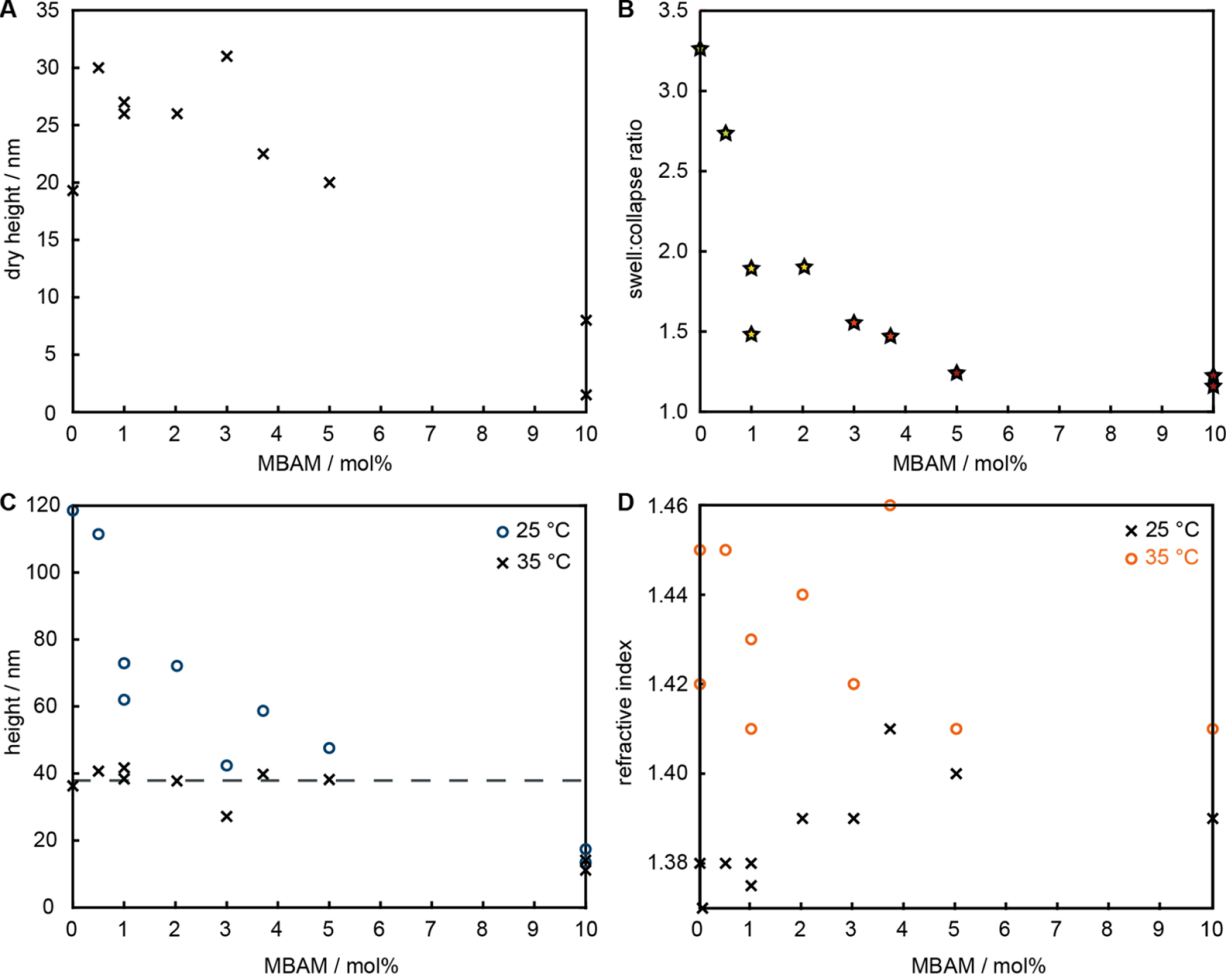
(A) Dry heights of PNIPAM-*co*-MBAM networks polymerized in 24 h (0.96 M, MeOH) decrease with increasing crosslinker content. (B) The swell/collapse height ratio decreases with increasing crosslinker content in the PNIPAM brushes. (C) Decrease in swollen height at 25 °C and constant collapsed height at 35 °C. (D) Refractive index of PNIPAM-*co*-MBAM networks with varying crosslinker content in PBS (pH 7.5) at 25 and 35 °C.

Crosslinked brushes with different fractions of MBAM were prepared in polymerizations of 24 h (the percentage crosslinker indicated is that from the reaction mixture, which we assume represents the percentage in the brush over these reaction durations). With a constant reaction time, the polymer network dry thickness decreased with increasing crosslinker content (Figure 2A). This is not unexpected, as crosslinkers are suspected to increase early termination reactions,^24^ and acrylic monomers can interact with the catalytic CuI/PMDETA complex, hindering ATRP.^51^

**Figure 3:**
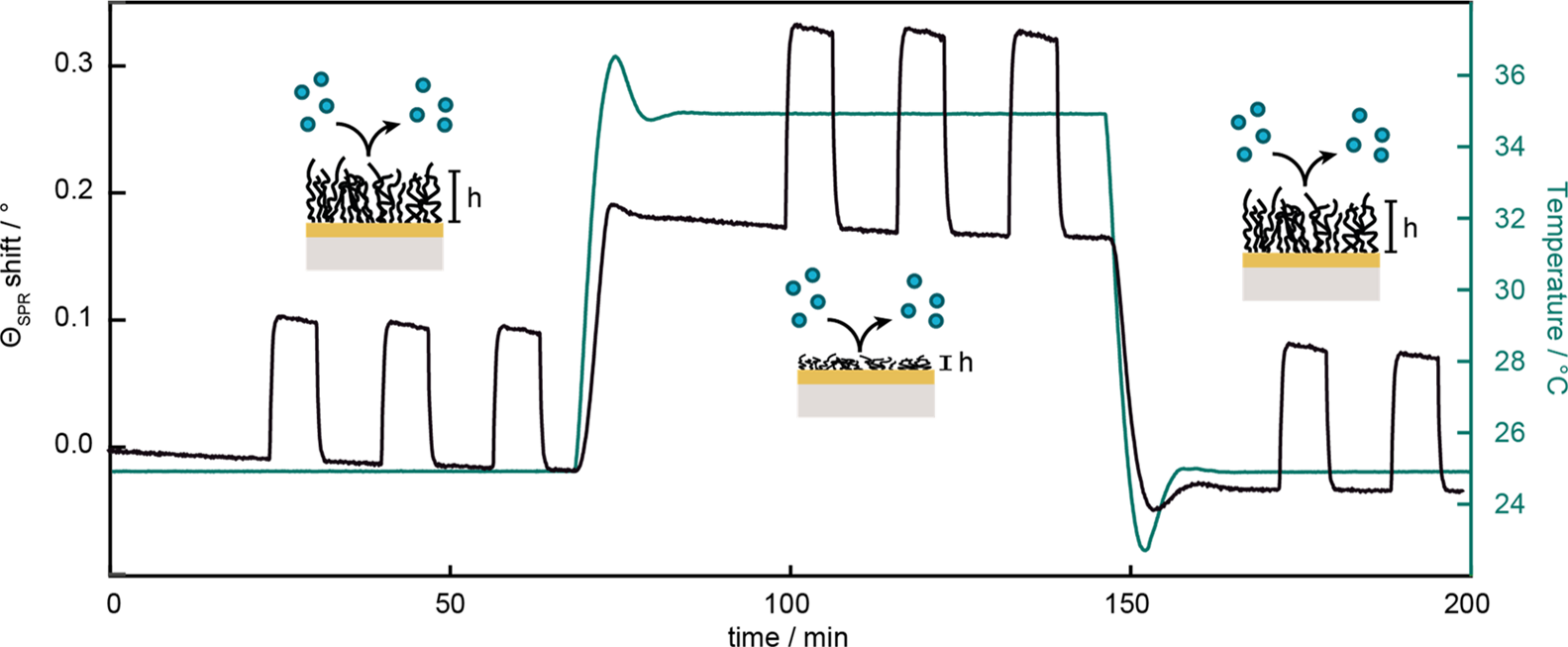
Non-interacting probe method in SPR using PEG (35 kDa, 10 mg mL^–1^) at 25 and 35 °C. PEG injections are indicated by reversible θ_SPR_ changes of ∼0.1°. θ_SPR_ increases with rising refractive index caused by a collapsing network at 35 °C and is fully reversible upon cooling.

Using polyethylene glycol (PEG) (35 kDa, 10 mg mL^–1^) as a non-interacting probe in SPR,^34^ we measured “swollen” and “collapsed” exclusion heights, that is, the characteristic height at which the probe molecules are expelled from the brush, of PNIPAM-*co*-MBAM brushes in PBS at 25 and 35 °C, respectively (Figure 3). The linear relation between the total internal reflection angle (which corresponds to bulk effects) and the SPR angle (which responds to both bulk and surface effects) confirms the non-interacting nature of PEG (Figure S4) and indicate that the change in SPR signal only corresponds to changes in the bulk refractive index.^44^ Collapsed gel networks were not free of water but contained roughly 45–50% by volume, calculated from the ratio of collapsed film thickness (in PBS at 35 °C) to dry film thickness in air. The ratio between swollen and collapsed heights, that is, the difference in wet height for the same surface at different temperatures (25 and 35 °C, respectively), is one important aspect of the thermoresponsive behavior of the PNIPAM networks, and how we define such behavior in this work. As this ratio approaches 1, thermoresponsive behavior is deemed to be lost. It was not observed to be a function of network height, but instead this ratio gradually decreased with increasing crosslinker content (Figure 2B). This is a direct consequence of decreasing swollen heights in more crosslinked networks as the collapsed heights remain constant (Figure 2C). Notably, the thermoresponsive behavior is almost completely lost in networks containing 5 mol % or more MBAM (swell/collapse factor ∼1.2) in a similar manner to PNIPAM-*co*-MBAM microgels deposited on solid substrates.^36^ The same trend is observed in the refractive indices: constant at 35 °C regardless of MBAM content (supporting that the networks are equally dense in the collapsed state), but increases at 25 °C in more crosslinked networks (Figure 2D). The lack of thermoresponsive behavior shown here is quite different from that reported for 5% ene-modified PNIPAM spin-coated and simultaneously crosslinked through thiol–ene click chemistry on a silica substrate (swelling factor ∼2.7 in water).^35^ Grafted-from gel networks are presumably influenced more by crosslinking as the applied shear in the spin-coating process might orient the networks preferentially in one direction^28^ and thereby reduce geometrical constraints that reduce the swollen thickness. Our crosslinked PNIPAM-*co*-MBAM_5%_ networks retained some hydrogel characteristic with swollen heights of approximately double the dry heights and ∼60 vol % water content above the VPTT (35 °C).

The reversible nature of the volume phase transition was confirmed by SPR (Figure 3). Upon expelling water when heating to 35 °C, the refractive index of the layer close to the surface increased, simultaneously causing a shift in θ_SPR_.^34^ Cooling back to 25 °C resulted in a reswelling of the gel to the same extent as before the first collapse, indicated by a shift in θ_SPR_ back to the original position.

**Figure 4:**
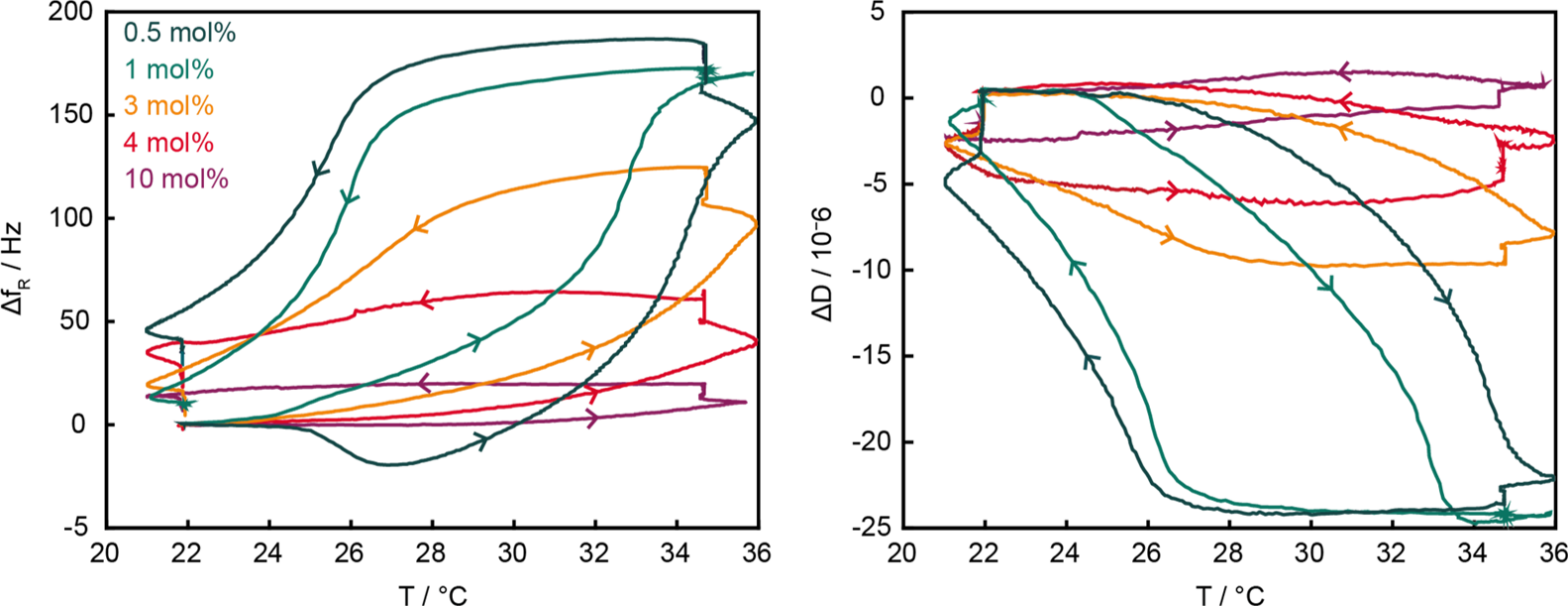
QCMD plot (left: resonance frequency, right: dissipation) of PNIPAM-*co*-MBAM with 0.5 mol % (dark green), 1 mol % (green), 3 mol % (orange), 4 mol % (red), and 10 mol % (purple) crosslinker content.

Integrated real-time temperature output with QCMD revealed further details on the temperature range in which the phase transition occurs. Upon heating to 35 °C, the networks expel water (*i.e.*, lose mass) and the resonance frequency increases. Simultaneously, the layer rigidifies, as evident by a decrease in dissipation (Figure 4). The phase transition is less pronounced in networks with a higher crosslinker content and is nonexistent in gels containing 10 mol % MBAM. The collapse takes place in a broad temperature regime between 27 and 34 °C for all crosslinker contents investigated (Figures 4 and S5). A different trend is visible in the reswelling of the layers upon cooling back to 22 °C: for networks containing up to 1 mol % MBAM, frequency and dissipation remained constant until 26 °C, followed by a sharp change within 2 °C, whereas more crosslinked layers transitioned gently within a 6 °C range (between 28 and 22 °C). Previously, differences between swelling and collapsing cycles were attributed to a “conformational memory effect”: inter- and intramolecular H-bonding in the collapsed state which suppresses rehydration.^52,53^ Crosslinking generally hinders hydration of surface-attached networks;^23−25^ thus, the gentle reswelling in our more crosslinked networks could result from a combination of a more pronounced memory effect and a slight VPTT increase in more crosslinked networks.^27^ Cycling between collapse and reswelling more slowly than the current rate (∼0.3 °C s^–1^) would likely decrease this hysteresis.^34^ Again, these results differ from the spin-coated PNIPAM gels (5% crosslinker) for which a sharp transition between 33 and 36 °C was observed.^35^

**Figure 5:**
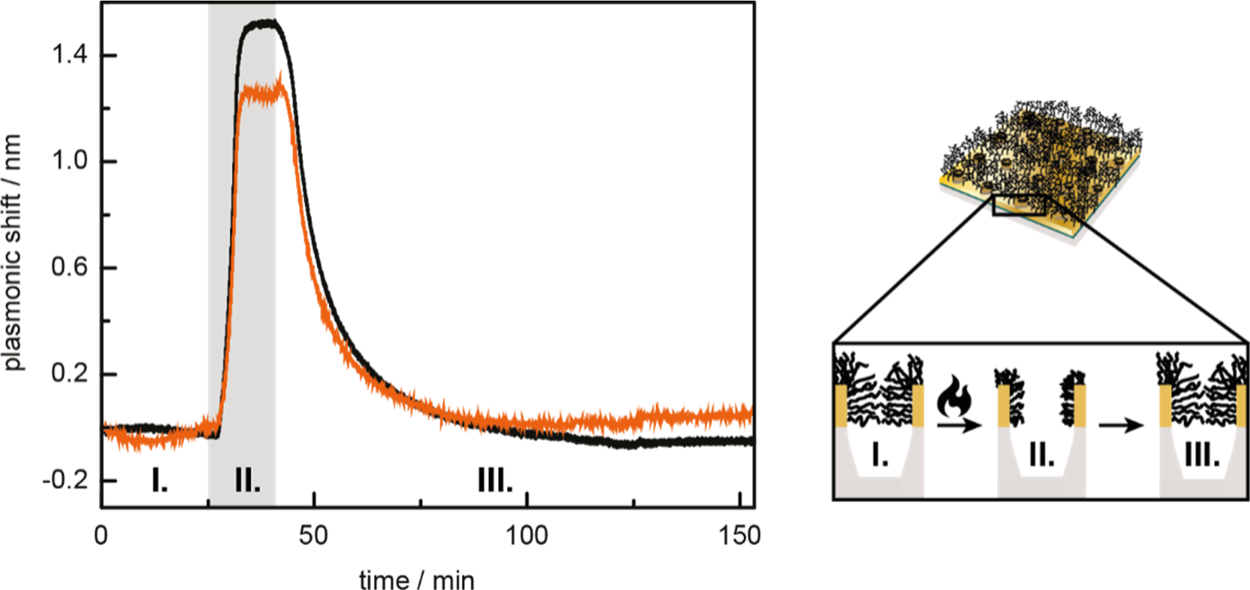
Monitoring shifts in peak (black) and dip (orange) position of a nanowell-patterned sensor coated with PNIPAM-*co*-MBAM_1%_ upon a temperature increase (35 °C, grey background) and subsequent decrease. The collapse and reswelling of the gel layer, opening and closing the pores respectively, is illustrated on the right.

Looking toward applications on nanostructured surfaces,^54^ we studied the behavior of PNIPAM-*co*-MBAM thermoresponsive gels on thin gold films containing plasmonic nanowell arrays (diameter: 90 nm) with optical properties discussed in detail in a previous report by our group.^55^ Briefly, such plasmonic structures display characteristic extinction spectra (absorption + scattering) with resonance features originating from the apertures in the thin metal film. Shifts in peak and dip position of the asymmetric resonance (Figure S6) correspond to refractive index changes on the surface and inside the nanowells, respectively.^55,56^ The thermoresponsive swelling behavior of our gel networks was retained on the patterned surfaces and mirrored that from the planar surfaces: more crosslinked networks swelled less (Figures 5 and S7). Thus, we propose that PNIPAM-*co*-MBAM brush networks are suitable for implementation on porous supports. They display tunable thermoresponsive behavior when surface confined and provide a logical next step beyond the PNIPAM brushes that have been investigated previously inside porous membranes.^57,58^

## Conclusions

In conclusion, PNIPAM-*co*-MBAM networks were synthesized *via* a simple and reliable SI-ARGET-ATRP strategy on gold films with thiol SAMs. At 0.96 M, the polymerization proceeded with fast reaction kinetics, giving networks with dry thicknesses >25 nm within 1 h. For better control over networks, the ATRP could be slowed by lowering the reaction concentration as demonstrated by an *in situ* QCMD experiment. The thermoresponsive phase transition of PNIPAM-*co*-MBAM gels is characterized by the swollen/collapsed height ratio. The ratio was not dependent on network height but could be tuned by varying the crosslinker content between 0 and 10 mol %. Investigations in SPR and QCMD showed that swollen gel heights (below VPTT) decreased with increasing crosslinker content, while collapsed heights (above VPTT) remained constant. This caused loss of thermoresponsiveness in gels containing at least 5 mol % MBAM, a significantly different behavior than observed in previous studies on spin-coated or bulk PNIPAM-based networks.^28,35,36,59^ A broader transition range was also evident for more crosslinked networks. In all, we have demonstrated a reliable method for tuning the swelling behavior of PNIPAM networks grafted from planar surfaces and then expanded this to patterned thin gold films containing nanowell arrays. By studying the behavior of PNIPAM-*co*-MBAM gels on nanostructured surfaces, we have paved the way for expanding their scope as thermo-responsive devices.

## Supporting Information Available

Method descriptions and spectra of IR-RAS, QCMD (with *in situ* polymerization), SPR, and LSPR; polymerization kinetic plot; SPR fitting parameters; film thicknesses obtained from SPR measurements; and estimated network water content (PDF)
