# Supplementary material for: Tuning the Thermoresponsive Behavior of Surface-Attached PNIPAM Networks: Varying the Crosslinker Content in SI-ATRP
Source: Langmuir. 2021 Mar 15;37(11):3391–8. doi: 10.1021/acs.langmuir.0c03545 (PMC8041372; doi:10.1021/acs.langmuir.0c03545)
Supplement: Supplementary file 1 — la0c03545_si_001.pdf [file la0c03545_si_001.pdf]

Tuning the Thermoresponsive Behavior of Surface-Attached PNIPAM Networks: Varying the Crosslinker Content in SI-ATRP

*Sophia Thiele, John Andersson, Andreas Dahlin, Rebekah L. N. Hailes**

* Corresponding author: rebekah@chalmers.se

Department of Chemistry and Chemical Engineering, Chalmers University of Technology, 41296 Gothenburg, Sweden.

TABLE OF CONTENTS

Measurement methods 3

Figures 6

Heights obtained from SPR measurements 10

References 11

Measurement methods

IR-RAS measurements: A Frontier FT-IR spectrometer (Perkin Elmer) equipped with a reflective absorbance sample holder was used to measure IR absorbance spectra of the PNIPAM-based networks. Background spectra were collected by scanning a bare gold SPR sensor. Three spectra were acquired in ten repeats between 400–4000 cm^{-1} on varied sensor surface positions and averaged.

QCMD measurements: QCMD sensors (diameter: 14 mm, f_R: 5 MHz) were from QuartzPro. Measurements were performed using a Q-Sense E4 (Biolin Scientific) equipped with a peristaltic pump. Ex-situ coated gold crystals were mounted in the measurement chamber and flushed with PBS until a stable baseline was established. The introduced error from subtracting f and D of a parallel measured reference were determined as ±5 Hz and $\pm 5 \times 10^{-6}$, respectively, by mounting clean gold sensors in the flow cells used and running the same temperature cycle. All data shown corresponds to the fifth overtone.

In-situ SI-ARGET-ATRP monitored in QCMD: Two reaction mixtures, complete and incomplete, were prepared according to a procedure described in the literature.[1] In one flask, monomer NIPAM (3.226 g, 28.5 mmol, 950 eq), crosslinker MBAM (44.4 mg, 0.288 mmol, 9.6 eq), and MeOH (30 mL) were added to inhibitor remover. The solution was degassed with N_2 for 5 min. In a second flask, $CuBr_2$ (6.7 mg, 0.030 mmol, 1 eq) was added to PMDETA (66.8 µL, 0.320 mmol, 10.7 eq) and 20 mL MeOH. 25 mL monomer solution was filtered (0.2 µm PTFE syringe filter) into the second flask, and the light blue solution obtained degassed for a further 20 min. For the incomplete reaction mixture, 5 mL monomer solution was added to a PMDETA solution (13.4 µL, 0.064 mmol, 2.1 eq) in 4 mL MeOH in a separate flask and degassed with N_2 for 20 min (no $CuBr_2$ was added). Separately, ascorbic acid (4.2 mg, 0.024 mmol, 0.8 eq) was added to MeOH (10 mL) and degassed for 20 min. 1 mL ascorbic

acid solution was added to the incomplete reaction mixture, and both solutions were kept under N_2 atmosphere throughout the whole experiment. An initiator functionalized QCMD sensor was mounted in the instrument, and the flow cell rinsed with the incomplete reaction mixture for 5 min at a flow rate of 166 μL min^{-1}. 5 mL ascorbic acid solution were added to the complete reaction mixture, and the blue solution became transparent. Finally, the flow was switched from the incomplete to complete reaction mixture using a valve and SI-ARGET-ATRP was monitored for 220 min. Voigt modelling and curve fitting was performed using the instrument specific software package Qtools using overtones 3, 5, 7 and 9.[2–5]

SPR measurements: Gold coated SPR sensors (50 nm Au on silica) were purchased from BioNavis. Measurements were performed on an SPR Navi 220A instrument (BioNavis) equipped with four lasers (2×670 nm, 785 nm and 980 nm) across two flow channels. The total internal reflection (TIR) and SPR angle were recorded in both air and buffer. The parameters used can be found in Table S1. Experiments in solution were conducted in PBS with a flow rate of 20 µL min^{-1}. After a stable baseline was established a non-interactive probe: PEG (35 kDa, 10 mg mL^{-1}), was injected in serial mode for 7 min, with a 7 min rinse with the running buffer in between each injection, and thereby spectra with and without the probe were collected. A linear relationship between shifts in θ_{SPR} and θ_{TIR} (Figure S4) confirms no interactions between the PNIPAM-co-MBAM networks and the PEG probe (35 kDa, 10 mg mL^{-1}). Background spectra of plain gold sensors in PBS (pH 7.5) were used to model the initial system without the polymer films. The bulk refractive index during probe injection was determined from shifts in θ_{TIR}. Fresnel models were performed for the spectra collected in air, and before, during, and after probe injection as described in previous reports.[6,7] Thereby, pairs of refractive index and thickness values were obtained, plotted, and the intersect revealed the correct thickness and refractive index.[6]

Table S1. Parameters used for Fresnel models for dry SPR spectra.

Layer	d [nm]	n 785 nm	κ 785 nm	n 670 nm	κ 670 nm
**silica/elastomer**	n.n.	1.5162	0	1.5020	0
**Cr**[6]	0.9	3.3225	3.6148	3.3105	3.4556
**Au**[8]	50	0.16088	5.0525	0.16194	3.9783
**Initiator**	2	1.45	0	1.45	0
**PNIPAM**[9]	h	1.4973	0.0018745	1.5	0.0021271
**air**	n.n.	1.0003	0	1.0003	0

Nanoplasmonic measurements: Measurements on nanowell patterned sensors (nanowell diameter: 90 nm) were carried out using an XNano flow cell (Insplorion, Sweden). The nanowells were prepared following a previously described procedure.[10] A peristaltic pump (Ismatec, Germany) pumped PBS (pH 7.5, 50 µL min^{-1}) through a metallic flow cell that provided for resistive heating. Extinction spectra were recorded, the peak and dip position selected, and changes monitored in a real-time measurement.

Figures

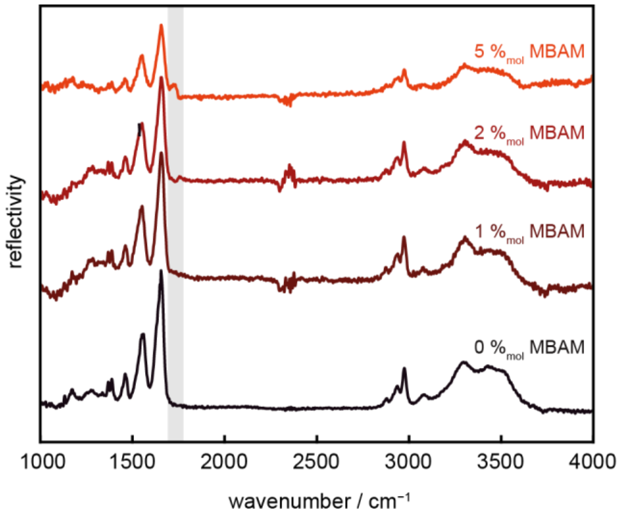

Figure S1. FT-IR spectra of PNIPAM-co-MBAM with increasing crosslinker content from bottom to top. The band (highlighted in grey) at 1725 cm^{-1} in gels containing more MBAM can be attributed to a C=O stretch.

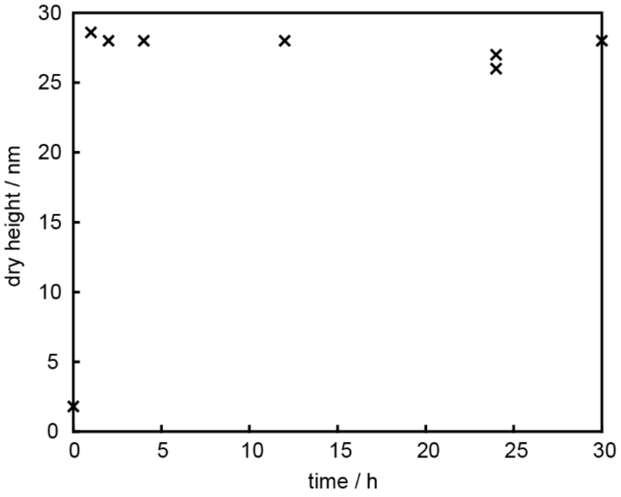

Figure S2. Kinetic plot of the polymerization of PNIPAM-co-MBAM containing 1 mol% crosslinker (MeOH, 0.96 M).

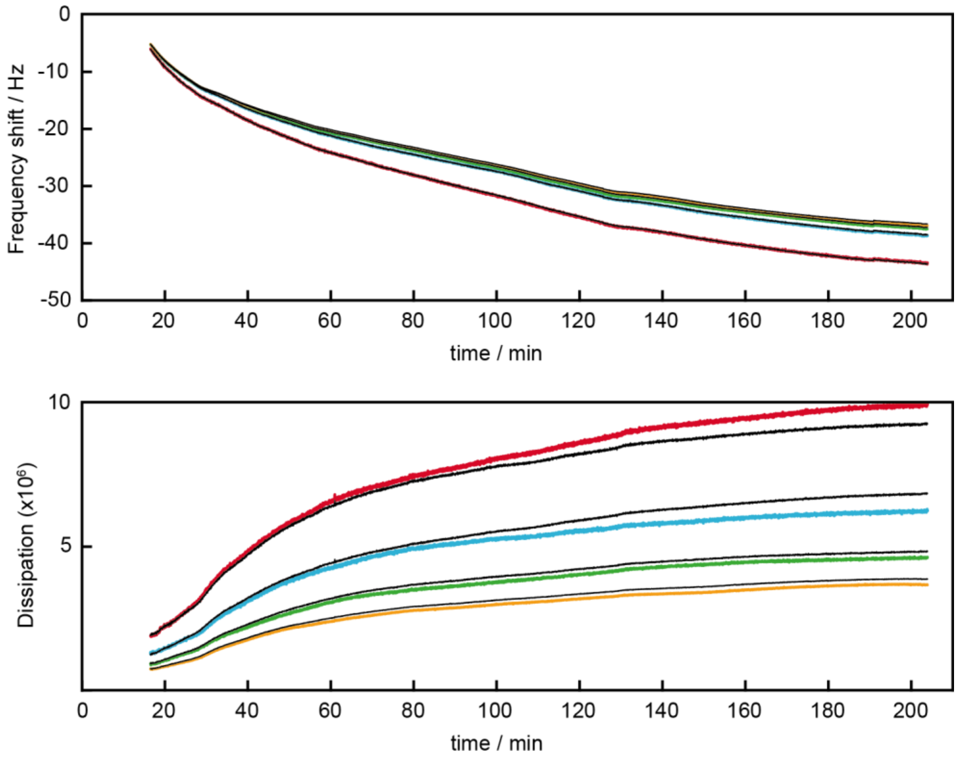

Figure S3. *In-situ* ATRP (MeOH, 0.48 м) monitored in QCMD yields PNIPAM-co-$MBAM_{1\%}$. Change in frequency and dissipation over time: overtone 3 (red), overtone 5 (blue), overtone 7 (green), overtone 9 (orange). Thinner black lines indicate fitted curves, which were used to calculate polymer network layer thickness (main text, Figure 1B).

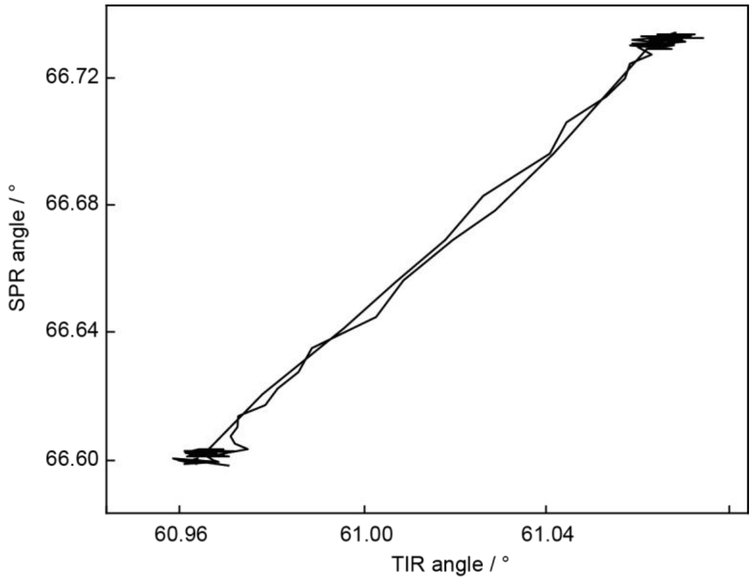

Figure S4. A linear relationship between the shift in θ_{SPR} and θ_{TIR} confirms no interactions between the PNIPAM-co-MBAM gels and PEG (35 kDa, 10 mg mL^{-1}).

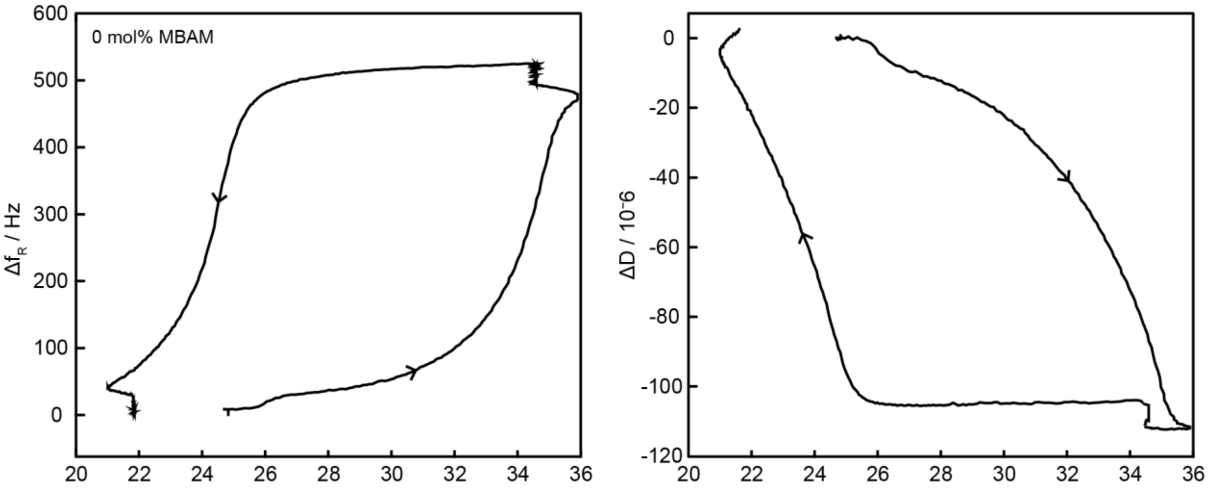

Figure S5. QCMD plot (left: resonance frequency, right: dissipation) of PNIPAM-co-MBAM with 0 mol% crosslinker content.

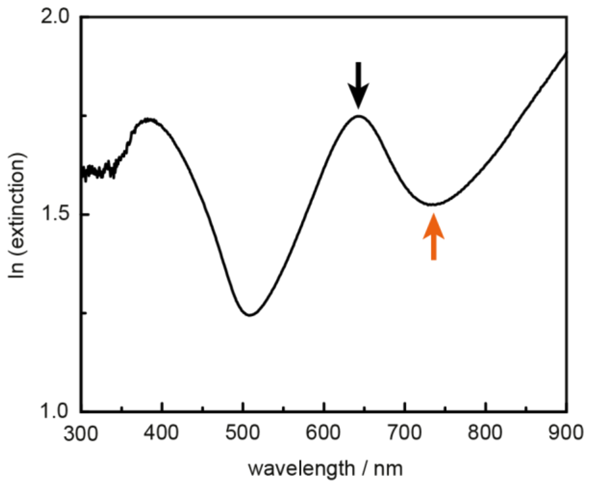

Figure S6. Characteristic peak (black arrow) and dip (orange arrow) are highlighted in the extinction spectrum of a nanowell patterned sensor coated with PNIPAM-co-MBAM$_{1\%}$.

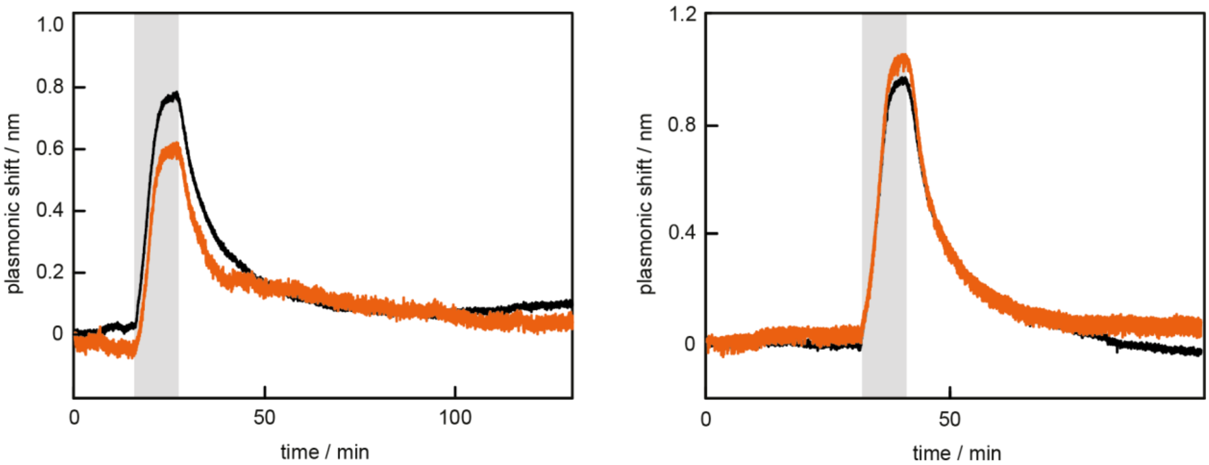

Figure S7. Shifts in peak (black) and dip (orange) position upon a temperature increase (35 °C, grey background) reveal a rise in the refractive index on top of the surface and inside the pores caused by a collapse of the PNIPAM-co-MBAM$_{4\%}$ (left) and PNIPAM-co-MBAM$_{2\%}$ (right) layer.

Heights obtained from SPR measurements

Table S2. Average heights obtained from SPR measurements, water content of swollen and collapsed networks, and calculated swell:collapse ratio. θ_{SPR} measured with two 670 nm lasers at different positions on one sample reveal a homogeneous layer thickness (Δdry_{max}=2.0 nm, $\Delta dry_{average}$=1.0 nm).

%mol MBAM	dry [nm]	Δdry [nm]	swollen [nm] /water content*	collapsed [nm] /water content*	swell:collapse ratio
0	19	0.6	119 / 84%	36 / 47%	3.27
0.5	30	1.3	112 / 73%	41 / 27%	2.74
1	27±1	1.4	68±5 / 60%	40±2 / 33%	1.70±0.2
2	26	0.2	72 / 64%	38 / 32%	1.91
3	31	2.0	42 / 26%	27 / −15%**	1.56
3.7	23	1.1	59 / 61%	40 / 43%	1.47
5	20	0.5	48 / 58%	38 / 47%	1.25
10	5±3	0.4	15±2 / 67%	13±1 / 62%	1.20±0.2

* estimated from swollen and collapsed film thicknesses.
** remaining water/air humidity in the “dry” sample might cause a thicker dry vs collapsed film.

References

[1] J. Mandal, R. S. Varunprasaath, W. Yan, M. Divandari, N. D. Spencer, M. Dübner, *RSC Advances* **2018**, *8*, 20048.

[2] M. V. Voinova, M. Rodahl, M. Jonson, B. Kasemo, *Phys. Scr.* **1999**, *59*, 391.

[3] F. Höök, B. Kasemo, T. Nylander, C. Fant, K. Sott, H. Elwing, *Anal. Chem.* **2001**, *73*, 5796.

[4] I. Reviakine, D. Johannsmann, R. P. Richter, *Anal. Chem.* **2011**, *83*, 8838.

[5] A. Saftics, G. A. Prósz, B. Türk, B. Peter, S. Kurunczi, R. Horvath, *Sci. Rep.* **2018**, *8*, 11840.

[6] G. Emilsson, R. L. Schoch, P. Oertle, K. Xiong, R. Y. H. Lim, A. B. Dahlin, *Appl. Surf. Sci.* **2017**, *396*, 384.

[7] G. Ferrand-Drake Del Castillo, G. Emilsson, A. Dahlin, *J. Phys. Chem. C* **2018**, *122*, 27516.

[8] D. I. Yakubovsky, A. V. Arsenin, Y. V. Stebunov, D. Yu. Fedyanin, V. S. Volkov, *Optics Express* **2017**, *25*, 25574.

[9] Y. Brasse, M. B. Müller, M. Karg, C. Kuttner, T. A. F. König, A. Fery, *ACS Appl. Mater. Interfaces* **2018**, *10*, 3133.

[10] B. Malekian, K. Xiong, G. Emilsson, J. Andersson, C. Fager, E. Olsson, E. M. Larsson-Langhammer, A. B. Dahlin, *Sensors* **2017**, *17*, 1444.
